# Supplementary material for: Transcriptional dysregulation by a nucleus-localized aminoacyl-tRNA synthetase associated with Charcot-Marie-Tooth neuropathy
Source: Nat Commun. 2019 Nov 6;10:5045. doi: 10.1038/s41467-019-12909-9 (PMC6834567; doi:10.1038/s41467-019-12909-9)
Supplement: Supplementary file 1 — Supplementary Information [file 41467_2019_12909_MOESM1_ESM.pdf]

Transcriptional dysregulation by a nucleus-localized aminoacyl-tRNA synthetase associated with Charcot-Marie-Tooth neuropathy

Bervoets, Wei et al.

Supplementary Information

Supplementary Fig. 1

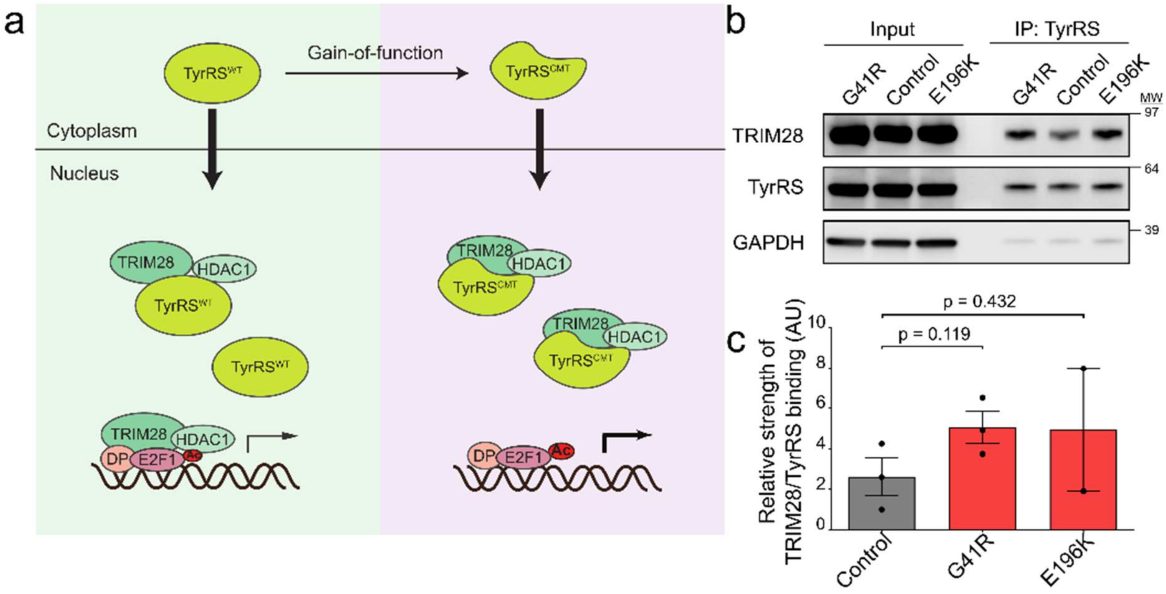

Supplementary Figure 1. Mutant TyrRS activates E2F1 in patient cells. (**a**) Schematic illustration of the mechanism through which CMT mutant TyrRS activates E2F1. Nuclear TRIM28 and HDAC1 are bound to the transcription factor E2F1 and its co-transcription factor DP, which are silenced because of HDAC1 deacetylation. In the mutant condition, TyrRS binds strongly to the TRIM28 and HDAC1 complex, releasing it from E2F1 and DP, as such activating the expression of E2F1 target genes. (**b**) Immunoprecipitation of endogenous TyrRS shows an interaction with endogenous TRIM28 in control and patients' PBMCs. (**c**) Quantification of the TRIM28-TyrRS interaction in patient derived PBMCs carrying the G41R (n=3 individuals) or E196K (n=2 individuals) mutation, compared to controls (n=3 individuals). The signal of co-purified TRIM28 was normalized to precipitated TyrRS. Unpaired t-test. Bar chart presented as means ± s.e.m. Source data are provided as a Source Data file.

Supplementary Fig. 2

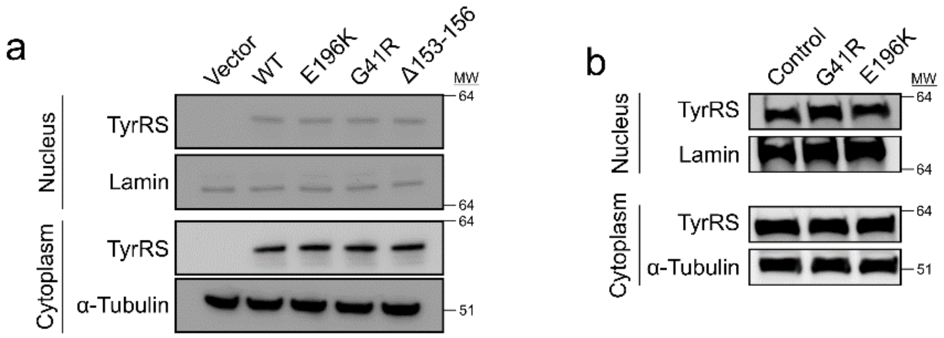

Supplementary Figure 2. Nuclear localisation of TyrRS in mammalian cells. (**a**) TyrRS localization in the cytoplasm and nucleus of HEK293T cells overexpressing different TyrRS alleles and (**b**) patient derived PBMCs endogenously expressing TyrRS. n=3 individual biological replicates. Source data are provided as a Source Data file.

Supplementary Fig. 3

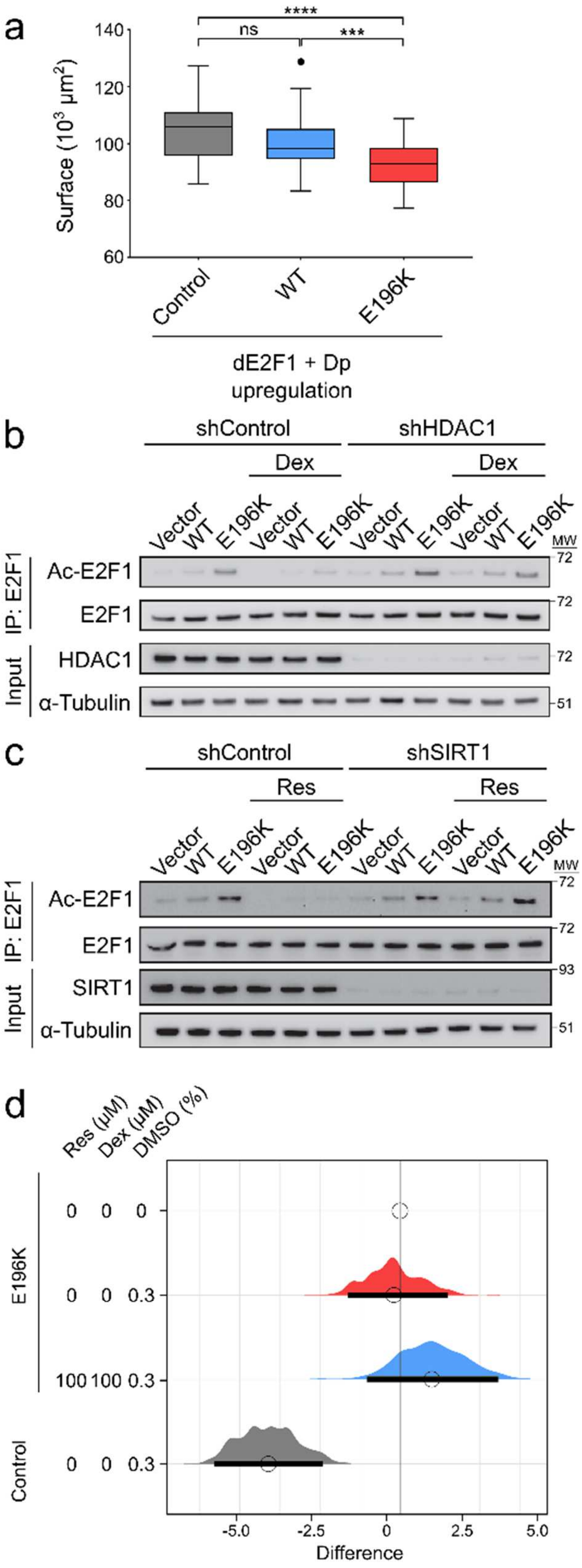

Supplementary Figure 3. Modulation of E2F1 activity. (**a**) Quantification of the rough eyes' size upon overexpression of dE2F1 and Dp in different genetic backgrounds. Box plots show the median, 25%-75% percentiles, and 1.5 interquartile range. n≥30 eyes. One-way ANOVA with Dunnetts Multicomparisons Test. (**b**) Targeting specificity of dexamethasone (Dex) on HDAC1 in HEK293T cells expressing various TyrRS genes; shHDAC1, short hairpin RNA targeting HDAC1. n=3 individual biological replicates. (**c**) Targeting specificity of resveratrol (Res) in HEK293T expressing various TyrRS genes; shSIRT1, short hairpin RNA targeting SIRT1. n=3 individual biological replicates. (**d**) Effect sizes of the climbing performance of flies pan-neuronally expressing (*nSyb-Gal4*) TyrRS-E196K when grown on food containing a combination of resveratrol and dexamethasone. n≥10 groups of flies. Horizontal bars depict the 95% confidence interval. Source data are provided as a Source Data file.

Supplementary Fig. 4

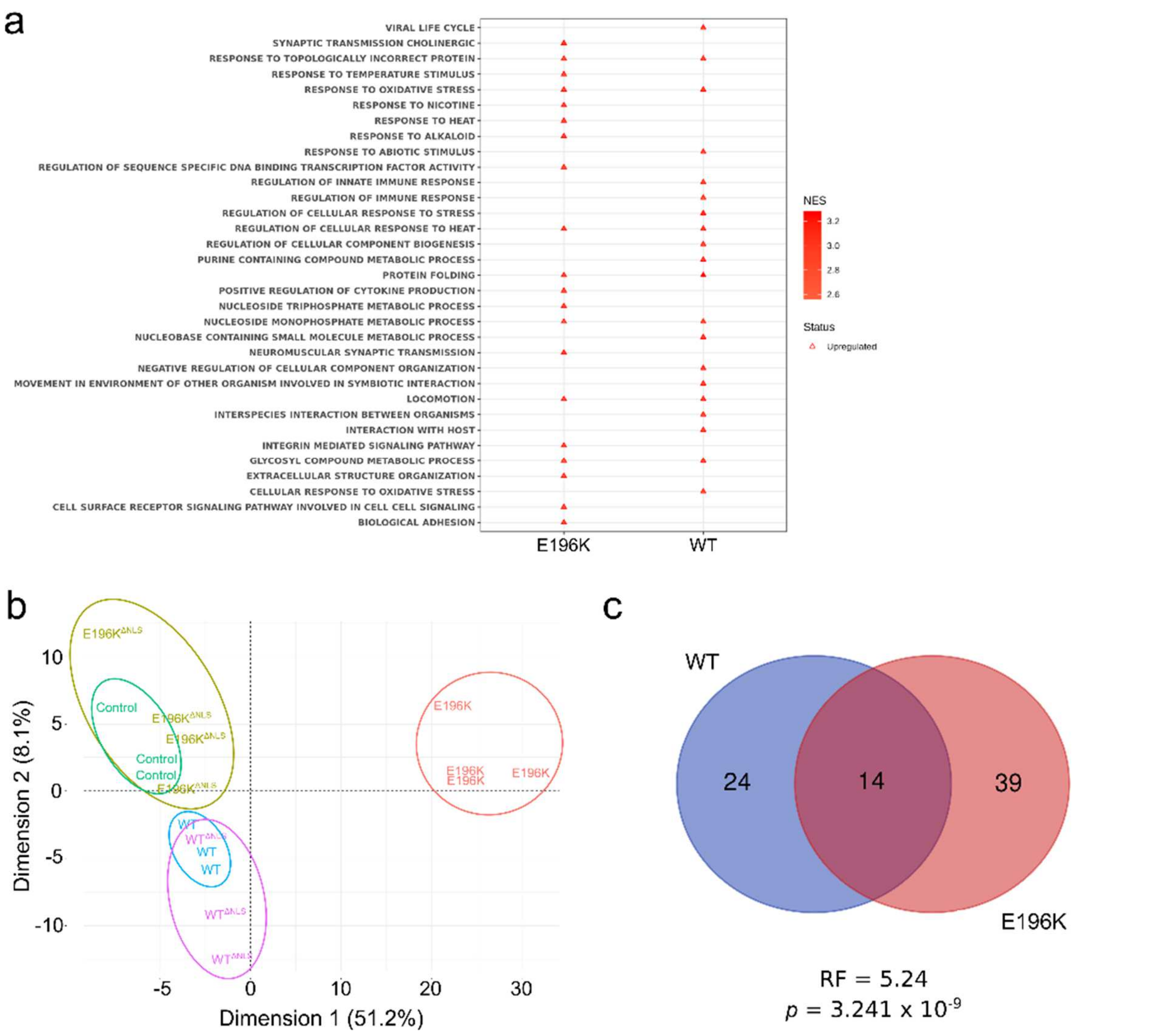

Supplementary Figure 4. Identification of a mutant specific gene cluster. (**a**) The plot shows the top 20 most enriched (NES) biological GO terms for each condition. Red triangles are overrepresented terms. The intensity of the colour defines the degree of overrepresentation. (**b**) PCA analysis of the cluster of 415 genes identified in control (*nSyb*>+), TyrRS-WT, TyrRS-WT$^{\Delta NLS}$, TyrRS-E196K, and TyrRS-E196K$^{\Delta NLS}$ expressing flies. The colours depict the different genotypes, while the circles show the confidence intervals. (**c**) Venn diagram depicting the overlap between the TFs identified in the TyrRS-WT and TyrRS-E196K conditions. RF, representation factor. *P*, probability.

Supplementary Fig. 5

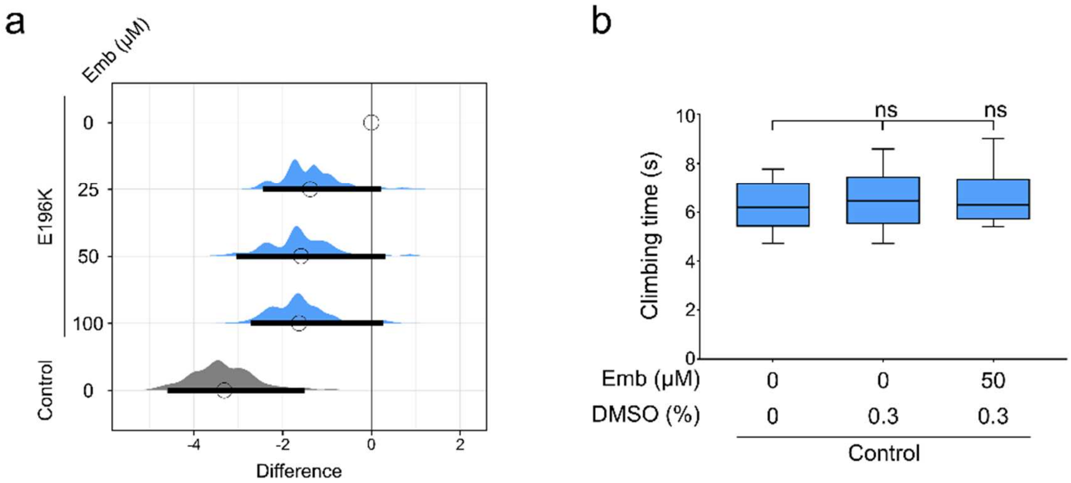

Supplementary Figure 5. Embelin treatment rescues disease phenotypes in a model for CMT. (**a**) Effect sizes of the climbing performance of flies pan-neuronally (*nSyb-Gal4*) expressing TyrRS-E196K raised on food containing embelin (Emb). n≥10 recordings. Horizontal bars depict the 95% confidence interval. (**b**) Climbing performance of control flies (*nSyb-Gal4>+*) raised on normal food, food containing 0.3% DMSO (42 mM), or 50 µM embelin in 0.3% DMSO (42 mM). n≥10 groups of flies. Unpaired t-test. Box plots show the median, 25%-75% percentiles, and 1.5 interquartile range. Source data are provided as a Source Data file.

Supplementary Fig. 6

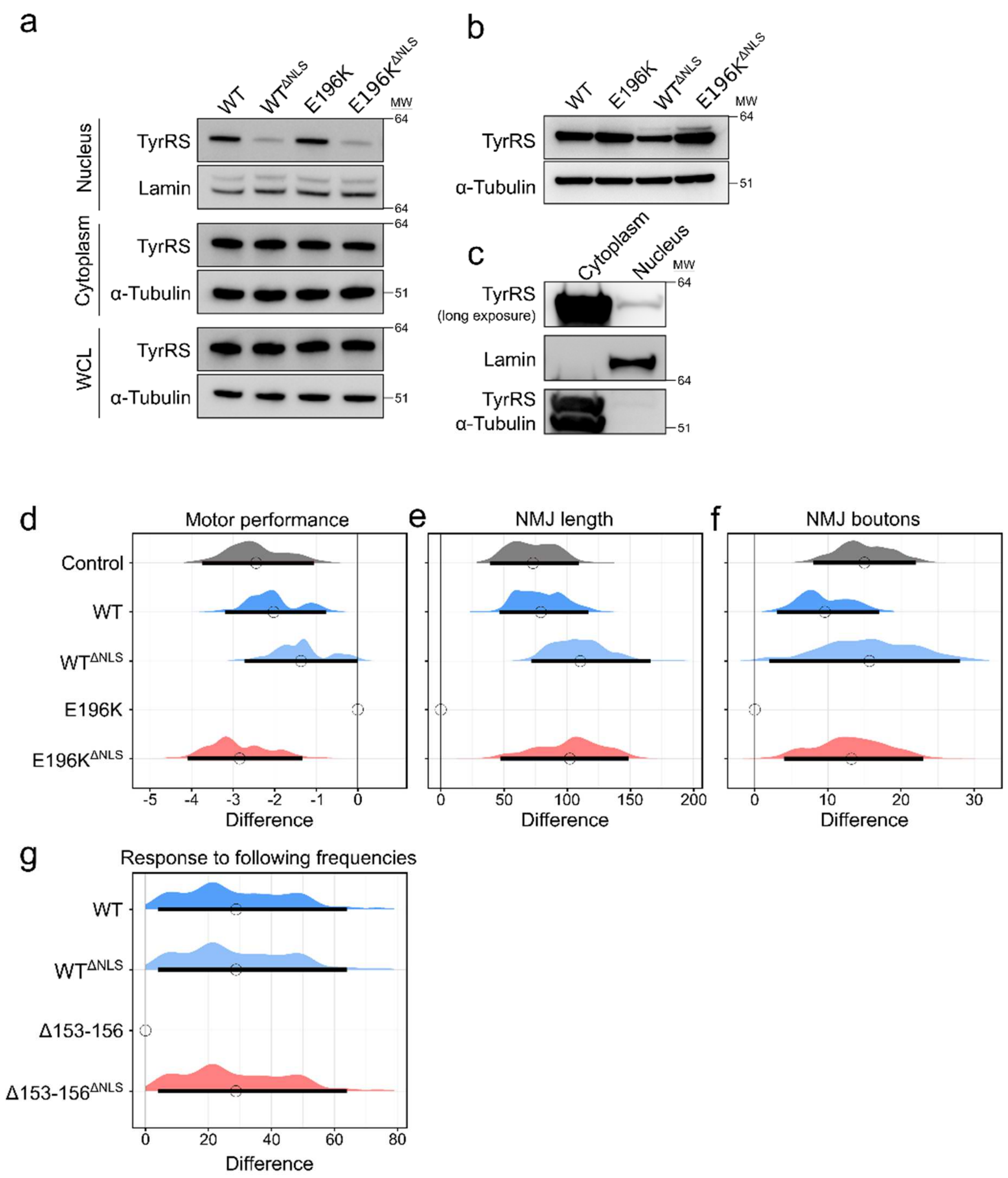

Supplementary Figure 6. Nuclear and extracellular localization of TyrRS. (**a**) Mutating the NLS sequence in TyrRS ($TyrRS^{\Delta NLS}$) reduces the nuclear localisation in HEK293T cells. (**b**) TyrRS expression levels in whole tissue lysate of heads taken from flies pan-neuronally (*nSyb-Gal4*) expressing different TyrRS alleles. The altered NLS still retains the acetylation site for PCAF (K242). Changing the surrounding basic residues from ^{242}KKKLKK^{247} to ^{242}NNKLNK^{247} may enhance acetylation but nevertheless disrupts nuclear translocation. We speculate that the higher molecular weight band is acetylated TyrRS which is unable to enter the nucleus (see also Fig. 5a). (**c**) Nuclear localization of human TyrRS in the brain of *Drosophila.* α-Tubulin and Lamin were used as controls for both the cytoplasmic and nuclear fractions. (**d**) Effect sizes of the climbing performance of flies pan-neuronally (*nSyb-Gal4*) expressing different TyrRS alleles ($n \geq 10$ groups of flies), as well as (**e**) NMJ length ($n \geq 10$ larvae), (**f**) total number of boutons ($n \geq 10$ larvae). (**g**) Effect sizes of the ability to follow repeated stimuli at 100 Hz upon presynaptic (*R91H05-Gal4*) expression of different TyrRS alleles in the giant fibre ($n \geq 20$ TTMs). Horizontal bars depict the 95% confidence interval. Source data are provided as a Source Data file.

Supplementary Fig. 7

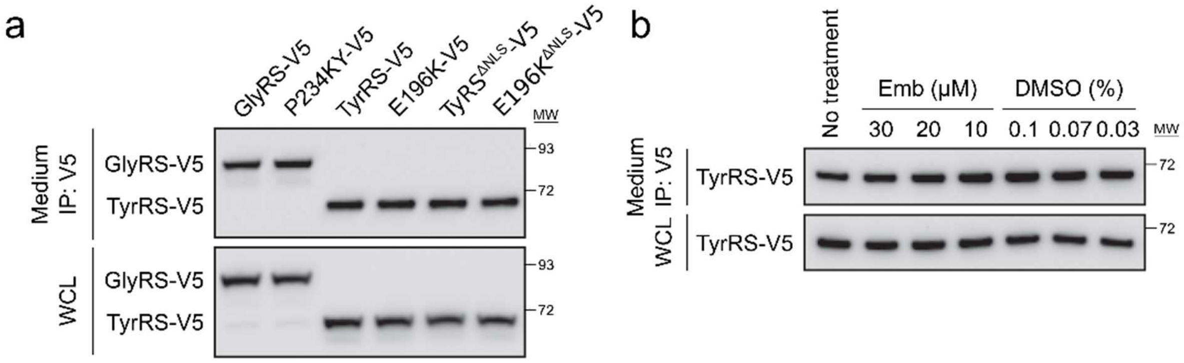

Supplementary Figure 7. Extracellular localization of TyrRS. (**a**) Neither the ΔNLS mutation nor the CMT mutation (E196K) affects the secretion of TyrRS from HEK293T cells. n=3 individual biological replicates. (**b**) Embelin treatment does not affect the secretion of TyrRS from HEK293T cells. n=3 individual biological replicates. Source data are provided as a Source Data file.

Supplementary Table 1. GO terms enriched in the TyrRS-WT brain transcriptome

pathway	pval	padj	ES	NES
protein folding	0.000186	0.001979	0.915594	3.27592
regulation of cellular response to stress	0.000181	0.001979	0.665344	2.969308
viral life cycle	0.000185	0.001979	0.81052	2.858782
negative regulation of cellular component organization	0.000187	0.001979	0.783739	2.843234
nucleobase containing small molecule metabolic process	0.000181	0.001979	0.707188	2.815087
purine containing compound metabolic process	0.000185	0.001979	0.796817	2.810451
locomotion	0.000177	0.001979	0.58513	2.777571
glycosyl compound metabolic process	0.000184	0.001979	0.732859	2.775165
regulation of cellular response to heat	0.000189	0.001979	0.953353	2.766729
response to abiotic stimulus	0.000179	0.001979	0.588885	2.762717
response to oxidative stress	0.000186	0.001979	0.77072	2.757575
interaction with host	0.000186	0.001979	0.821405	2.745016
movement in environment of other organism involved in symbiotic interaction	0.000188	0.001979	0.840717	2.73995
regulation of cellular component biogenesis	0.000181	0.001979	0.686154	2.73136
nucleoside monophosphate metabolic process	0.000188	0.001979	0.857728	2.721088
response to topologically incorrect protein	0.000187	0.001979	0.798428	2.718921
interspecies interaction between organisms	0.000182	0.001979	0.6473	2.713604
regulation of innate immune response	0.000188	0.001979	0.822985	2.682161
regulation of apoptotic signaling pathway	0.000187	0.001979	0.769614	2.667114
cellular response to oxidative stress	0.000188	0.001979	0.839877	2.664457
regulation of intracellular transport	0.000182	0.001979	0.663423	2.657443
regulation of immune response	0.000183	0.001979	0.626814	2.642253
nucleoside triphosphate metabolic process	0.000189	0.001979	0.905799	2.628723
negative regulation of apoptotic signaling pathway	0.000188	0.001979	0.803049	2.617188
regulation of protein stability	0.000189	0.001979	0.842966	2.60664
regulation of protein modification by small protein conjugation or removal	0.000189	0.001979	0.839912	2.597197
organophosphate metabolic process	0.000179	0.001979	0.552876	2.59378
regulation of organelle organization	0.000183	0.001979	0.615158	2.593119
positive regulation of cytokine production	0.000187	0.001979	0.747065	2.588971
positive regulation of sequence specific dna binding transcription factor activity	0.000189	0.001979	0.836591	2.586928
regulation of sequence specific dna binding transcription factor activity	0.000187	0.001979	0.746185	2.58592
regulation of response to stress	0.000174	0.001979	0.509312	2.584051
positive regulation of nf kappab transcription factor activity	0.00019	0.001979	0.86032	2.578492
negative regulation of protein modification process	0.000182	0.001979	0.595183	2.573752
cellular response to stress	0.000175	0.001979	0.492046	2.54659
positive regulation of immune system process	0.000182	0.001979	0.611083	2.5397
regulation of defense response	0.000182	0.001979	0.632831	2.534901
regulation of extrinsic apoptotic signaling pathway	0.00019	0.001979	0.844031	2.529669
regulation of cytokine production	0.000184	0.001979	0.667971	2.529451
carbohydrate derivative metabolic process	0.000175	0.001979	0.496212	2.428891
regulation of immune system process	0.000179	0.001979	0.521947	2.407745
negative regulation of cell death	0.000181	0.001979	0.538211	2.386464
positive regulation of intracellular signal transduction	0.000181	0.001979	0.52429	2.376241
mitochondrion organization	0.000185	0.001979	0.63441	2.37135
regulation of cellular localization	0.000178	0.001979	0.499839	2.358642
integrin mediated signaling pathway	0.000372	0.003623	0.643664	2.302979
negative regulation of cell communication	0.000177	0.001979	0.474043	2.256679
negative regulation of intracellular transport	0.000568	0.005349	0.76576	2.222314
positive regulation of gene expression	0.000175	0.001979	0.445661	2.181449
regulation of transport	0.000173	0.001979	0.414103	2.161893
regulation of cell death	0.000175	0.001979	0.43587	2.155957
positive regulation of multicellular organismal process	0.000355	0.003516	0.450231	2.152064
regulation of intracellular signal transduction	0.000174	0.001979	0.426078	2.151723
positive regulation of establishment of protein localization	0.001304	0.011712	0.643175	2.1494
positive regulation of cell communication	0.000174	0.001979	0.413295	2.117931
negative regulation of response to stimulus	0.000176	0.001979	0.425999	2.099468
anatomical structure homeostasis	0.002992	0.022695	0.609033	2.073968

regulation of cellular protein localization	0.002607	0.020575	0.61951	2.070313
positive regulation of cellular protein localization	0.003201	0.023966	0.651446	2.066674
positive regulation of intracellular transport	0.001898	0.015778	0.689257	2.065791
dna repair	0.001811	0.015556	0.518848	2.065368
negative regulation of intracellular signal transduction	0.002801	0.021807	0.558181	2.05544
regulation of intracellular protein transport	0.001893	0.015778	0.704406	2.04426
positive regulation of biosynthetic process	0.000349	0.003513	0.405074	2.036267
positive regulation of organelle organization	0.003722	0.027517	0.567611	2.030868
posttranscriptional regulation of gene expression	0.004483	0.03154	0.585867	2.030335
phosphate containing compound metabolic process	0.000171	0.001979	0.377274	2.028116
regulation of cytoplasmic transport	0.003926	0.02866	0.558785	2.027149
sexual reproduction	0.005397	0.037083	0.557749	1.99558
positive regulation of molecular function	0.000173	0.001979	0.374551	1.987173
negative regulation of transport	0.002915	0.022403	0.484624	1.977694
positive regulation of response to stimulus	0.000173	0.001979	0.36849	1.952936
regulation of protein modification process	0.000173	0.001979	0.369408	1.938571
organonitrogen compound metabolic process	0.00052	0.004975	0.364133	1.929845
regulation of protein localization	0.005098	0.035445	0.472983	1.912226
negative regulation of protein metabolic process	0.001732	0.015096	0.352082	1.856882
positive regulation of transport	0.00639	0.041466	0.433768	1.844904
chromosome organization	0.00639	0.041466	0.43339	1.843295
cellular response to dna damage stimulus	0.007303	0.045861	0.429494	1.826724
positive regulation of hydrolase activity	0.006684	0.042892	0.399185	1.789283
vesicle mediated transport	0.005969	0.040066	-0.33742	-1.71455
organic anion transport	0.006769	0.042966	-0.4573	-1.85924
regulation of exocytosis	0.007447	0.046265	-0.58199	-1.87552
lipid catabolic process	0.006074	0.040308	-0.51014	-1.88374
aminoglycan biosynthetic process	0.005705	0.038738	-0.61965	-1.93166
developmental process involved in reproduction	0.004088	0.029113	-0.53252	-1.93344
ion transport	0.001163	0.01061	-0.3754	-1.94196
regulation of receptor activity	0.004049	0.029113	-0.58796	-1.94586
regulated exocytosis	0.002367	0.018941	-0.54839	-1.99104
secretion by cell	0.002368	0.018941	-0.52013	-2.02612
cellular lipid catabolic process	0.001918	0.015778	-0.61499	-2.0353
secretion	0.001339	0.011848	-0.48997	-2.05889
exocytosis	0.00108	0.010013	-0.56084	-2.11411
bicarbonate transport	0.000211	0.002165	-0.78497	-2.44701

ES, Enrichment Score

NES, Normalized Enrichment Score

Supplementary Table 2. Transcription factors enriched in the TyrRS-WT brain transcriptome, in both up-and down-regulated gene lists.

Transcription factor	Gene symbol	E-score	Targets	Motifs/Tracks
**Downregulated gene list**				
grain	grn	4.741	120	32
longitudinals lacking	lola	4.521	47	5
gemini	gem	4.192	94	6
TBP-associated factor 1	Taf1	3.735	51	1
CG9727	CG9727	3.726	56	6
Hormone receptor 3	Hr46	3.699	31	1
Dorsal root ganglia homeobox	CG34340	3.317	50	6
bigmax	bigmax	3.246	57	2
glass	gl	3.209	55	4
maternal gene required for meiosis	mamo	3.196	32	3
Forkhead box K	fd68A	3.180	40	3
Jun-related antigen	Jra	3.164	94	5
Myocyte enhancer factor 2	Mef2	3.084	44	3
Cyclic-AMP response element binding protein B	CrebB-17A	3.020	65	2
pleiohomeotic	pho	2.883	17	1
proboscipedia	pb	2.843	29	2
Kruppel	Kr	2.695	24	1
glial cells missing	gcm	2.627	11	1
hairy	h	2.615	63	2
forkhead box, sub-group O	foxo	2.544	20	1
**Upregulated gene list**				
Rel	Rel	5.600	122	23
Activating transcription factor 3	Atf3	5.214	124	55
grain	grn	4.649	186	22
Cyclic-AMP response element binding protein B	CrebB-17A	3.967	77	12
Ecdysone receptor	EcR	3.636	58	3
TBP-associated factor 1	Taf1	3.534	68	2
E2F transcription factor 1	E2f	3.496	32	3
cap-n-collar	cnc	3.360	63	3
extra macrochaetae	emc	3.350	34	2
vrille	vri	3.284	41	5
Myocyte enhancer factor 2	Mef2	3.094	76	8
Atf6	Atf6	3.085	59	3
Forkhead box K	fd68A	3.071	29	2
ken and barbie	ken	3.046	69	8
Sp1	Sp1	2.929	24	2
pannier	pnr	2.864	29	2
grainy head	grh	2.692	85	3
Heat shock factor	Hsf	2.665	52	2
lame duck	lmd	2.642	20	2
sidestep	Side	2.604	51	2
Rfx	Rfx	2.595	12	1
Sox100B	Sox100B	2.557	14	1

E-score, enrichment score

Supplementary Table 3. GO terms enriched in the TyrRS-E196K brain transcriptome

pathway	pval	padj	ES	NES
neuromuscular synaptic transmission	0.000191	0.00316	0.667553	2.869348
synaptic transmission cholinergic	0.000191	0.00316	0.667553	2.869348
regulation of cellular response to heat	0.000195	0.00316	0.721983	2.830654
integrin mediated signaling pathway	0.000195	0.00316	0.790135	2.816679
response to nicotine	0.00019	0.00316	0.616832	2.810326
response to heat	0.000194	0.00316	0.855255	2.761712
protein folding	0.000188	0.00316	0.526361	2.736879
response to temperature stimulus	0.000195	0.00316	0.76746	2.735845
nucleoside triphosphate metabolic process	0.000195	0.00316	0.69798	2.680139
regulation of sequence specific dna binding transcription factor activity	0.000195	0.00316	0.747169	2.663513
biological adhesion	0.000186	0.00316	0.468612	2.651161
response to alkaloid	0.000188	0.00316	0.547559	2.630954
cell surface receptor signaling pathway involved in cell cell signaling	0.00019	0.00316	0.57704	2.629027
nucleoside monophosphate metabolic process	0.000192	0.00316	0.653991	2.623176
positive regulation of cytokine production	0.000192	0.00316	0.651962	2.615036
response to oxidative stress	0.000191	0.00316	0.59135	2.607821
response to topologically incorrect protein	0.000195	0.00316	0.778937	2.604571
regulation of protein modification by small protein conjugation or removal	0.000191	0.00316	0.603022	2.591972
glycosyl compound metabolic process	0.000192	0.00316	0.613047	2.58845
locomotion	0.000183	0.00316	0.427917	2.580618
regulation of apoptotic signaling pathway	0.000192	0.00316	0.610933	2.579526
extracellular structure organization	0.000189	0.00316	0.542674	2.578641
negative regulation of apoptotic signaling pathway	0.000195	0.00316	0.686541	2.57623
regulation of cytokine production	0.00019	0.00316	0.559582	2.549489
muscle contraction	0.000191	0.00316	0.581603	2.527428
negative regulation of protein modification by small protein conjugation or removal	0.000194	0.00316	0.781924	2.524917
cellular response to oxidative stress	0.000195	0.00316	0.753428	2.519275
muscle system process	0.000191	0.00316	0.567294	2.501735
cell matrix adhesion	0.000195	0.00316	0.684635	2.501488
regulation of response to cytokine stimulus	0.000194	0.00316	0.771766	2.492116
rrna metabolic process	0.000374	0.004971	0.471834	2.491509
regulation of immune response	0.000187	0.00316	0.496588	2.478987
purine containing compound metabolic process	0.000191	0.00316	0.562126	2.478944
regulation of innate immune response	0.000193	0.00316	0.607693	2.476902
movement in environment of other organism involved in symbiotic interaction	0.000195	0.00316	0.730945	2.444097
ribosome biogenesis	0.000374	0.004971	0.439829	2.440281
regulation of mitochondrion organization	0.000195	0.00316	0.701785	2.42838
regulation of cellular response to stress	0.000374	0.004971	0.457624	2.41647
regulation of extrinsic apoptotic signaling pathway	0.000195	0.00316	0.718662	2.403026
interaction with host	0.000195	0.00316	0.657118	2.40095
translational initiation	0.000193	0.00316	0.577876	2.393706
regulation of protein stability	0.000195	0.00316	0.669358	2.386131
positive regulation of immune system process	0.000565	0.006366	0.45541	2.385259
regulation of immune system process	0.00037	0.004971	0.405488	2.349494
cell substrate adhesion	0.000195	0.00316	0.599125	2.348967
locomotory behavior	0.000384	0.004971	0.585011	2.346496
negative regulation of cell death	0.000374	0.004971	0.426703	2.325219
negative regulation of protein modification process	0.000564	0.006366	0.452559	2.317243
establishment of protein localization to endoplasmic reticulum	0.000384	0.004971	0.576855	2.313781
cell motility	0.000374	0.004971	0.416973	2.313467
adult behavior	0.00039	0.004971	0.612725	2.299237
synaptic signaling	0.000563	0.006366	0.478037	2.296912
chloride transport	0.000388	0.004971	0.705657	2.278641
inorganic anion transport	0.000388	0.004971	0.705657	2.278641
protein localization to membrane	0.00057	0.006366	0.478577	2.250402

establishment of protein localization to membrane	0.000576	0.006366	0.53196	2.246081
protein targeting to membrane	0.000576	0.006366	0.53196	2.246081
regulation of membrane potential	0.000955	0.009455	0.514068	2.233948
nuclear transcribed mrna catabolic process nonsense mediated decay	0.001157	0.01065	0.536034	2.220385
behavior	0.000751	0.007658	0.461396	2.216954
protein localization to endoplasmic reticulum	0.001156	0.01065	0.531599	2.166748
system process	0.000179	0.00316	0.332111	2.163896
nucleobase containing small molecule metabolic process	0.001119	0.01065	0.427254	2.153488
single organism behavior	0.00192	0.016977	0.508368	2.146469
regulation of cell death	0.00055	0.006366	0.353846	2.139163
regulation of cellular component biogenesis	0.001524	0.013845	0.458309	2.131525
organophosphate metabolic process	0.000745	0.007658	0.374905	2.110485
response to abiotic stimulus	0.000728	0.007658	0.340591	2.109951
positive regulation of intracellular signal transduction	0.002252	0.019393	0.436916	2.09933
positive regulation of molecular function	0.000545	0.006366	0.326942	2.072152
cell cell signaling	0.001125	0.01065	0.386857	2.069431
regulation of defense response	0.002612	0.021158	0.405535	2.062454
ribonucleoprotein complex biogenesis	0.001116	0.01065	0.362227	2.059418
viral life cycle	0.002073	0.018085	0.399088	2.059328
regulation of rna stability	0.00293	0.022586	0.593648	2.054195
skeletal muscle organ development	0.002729	0.021287	0.5466	2.051101
muscle organ development	0.002699	0.021287	0.490917	2.033502
muscle structure development	0.002662	0.021265	0.439582	2.021794
regulation of response to stress	0.000726	0.007658	0.319518	2.016673
carbohydrate derivative metabolic process	0.002604	0.021158	0.35145	1.998148
ncrna processing	0.002617	0.021158	0.356047	1.994618
cellular response to stress	0.000899	0.009029	0.304947	1.980516
response to endogenous stimulus	0.000717	0.007658	0.302634	1.971834
positive regulation of multicellular organismal process	0.001653	0.014814	0.329576	1.962972
regulation of intracellular transport	0.004944	0.03602	0.415963	1.955975
leukocyte migration	0.00547	0.038174	0.497239	1.949507
muscle cell development	0.006623	0.042219	0.530265	1.937458
regulation of anion transport	0.007406	0.044638	0.541904	1.931784
muscle tissue development	0.006336	0.040787	0.477705	1.916089
negative regulation of response to stimulus	0.002569	0.021158	0.319252	1.912638
response to nitrogen compound	0.005018	0.03616	0.330144	1.899439
transmembrane transport	0.003084	0.023499	0.29932	1.889192
regulation of ion transport	0.007132	0.044193	0.391979	1.883412
cellular response to lipid	0.006203	0.040785	0.581742	1.878508
circulatory system process	0.008217	0.049076	0.431824	1.876546
negative regulation of cell communication	0.005362	0.037822	0.323326	1.873426
immune system process	0.003431	0.025558	0.28875	1.85615
negative regulation of cellular component organization	0.007332	0.044599	0.361639	1.851705
membrane organization	0.006916	0.043257	0.329866	1.847947
positive regulation of response to stimulus	0.004739	0.034913	0.292654	1.82159
carbohydrate metabolic process	0.005675	0.03919	0.29652	1.813848
positive regulation of gene expression	0.005076	0.036188	0.282459	1.797109
ion transmembrane transport	0.007201	0.044205	0.301663	1.789553
positive regulation of cell communication	0.00829	0.049076	0.305043	1.778672
regulation of transport	0.006252	0.040785	0.269694	1.778518
positive regulation of biosynthetic process	0.006275	0.040785	0.272493	1.77545
interspecies interaction between organisms	0.008523	0.049569	0.301766	1.771992
phosphate containing compound metabolic process	0.006724	0.04246	0.256848	1.751409
response to organic cyclic compound	0.00842	0.049404	0.284458	1.740063
chromatin assembly or disassembly	0.008621	0.0497	-0.48811	-1.87239
negative regulation of peptidase activity	0.006241	0.040785	-0.36207	-1.87281
small gtpase mediated signal transduction	0.006158	0.040785	-0.4877	-1.92174
dna packaging	0.006158	0.040785	-0.48854	-1.92503
bicarbonate transport	0.003303	0.024886	-0.61316	-1.99763
neutral lipid metabolic process	0.002457	0.020885	-0.59745	-2.01706

ES, Enrichment Score
NES, Normalized Enrichment Score

Supplementary Table 4. Overlap GO terms between the E196K and WT brain transcriptomes

	NES	
**Biological process**	**E196K**	**WT**
adult behavior	2.3**	
aminoglycan biosynthetic process		-1.9*
anatomical structure homeostasis		2.1*
behavior	2.2**	
bicarbonate transport	-2.0*	-2.4**
biological adhesion	2.7**	
carbohydrate derivative metabolic process	2.0*	2.4**
carbohydrate metabolic process	1.8*	
cellular lipid catabolic process		-2.0*
cellular response to dna damage stimulus		1.8*
cellular response to lipid	1.9*	
cellular response to oxidative stress	2.5**	2.7**
cellular response to stress	2.0**	2.5**
cell cell signaling	2.1*	
cell matrix adhesion	2.5**	
cell motility	2.3**	
cell substrate adhesion	2.3**	
cell surface receptor signaling pathway involved in cell cell signaling	2.6**	
chloride transport	2.3**	
chromatin assembly or disassembly	-1.9*	
chromosome organization		1.8*
circulatory system process	1.9*	
developmental process involved in reproduction		-1.9*
dna packaging	-1.9*	
dna repair		2.1*
establishment of protein localization to endoplasmic reticulum	2.3**	
establishment of protein localization to membrane	2.2**	
exocytosis		-2.1*
extracellular structure organization	2.6**	
glycosyl compound metabolic process	2.6**	2.8**
immune system process	1.9*	
inorganic anion transport	2.3**	
integrin mediated signaling pathway	2.8**	2.3**
interaction with host	2.4**	2.7**
interspecies interaction between organisms	1.8*	2.7**
ion transmembrane transport	1.8*	
ion transport		-1.9*
leukocyte migration	1.9*	
lipid catabolic process		-1.9*
locomotion	2.6**	2.8**
locomotory behavior	2.3**	
membrane organization	1.8*	1.7
mitochondrion organization		2.4**
movement in environment of other organism involved in symbiotic interaction	2.4**	2.7**
muscle cell development	1.9*	
muscle contraction	2.5**	
muscle organ development	2.0*	
muscle structure development	2.0*	
muscle system process	2.5**	
muscle tissue development	1.9*	
ncrna processing	2.0*	
negative regulation of apoptotic signaling pathway	2.6**	2.6**
negative regulation of cellular component organization	1.9*	2.8**
negative regulation of cell communication	1.9*	2.3**
negative regulation of cell death	2.3**	2.4**
negative regulation of intracellular signal transduction		2.1*
negative regulation of intracellular transport		2.2**
negative regulation of peptidase activity	-1.9*	
negative regulation of protein modification by small protein conjugation or removal	2.5**	

negative regulation of protein modification process	2.3**	2.6**
negative regulation of response to stimulus	1.9*	2.1**
negative regulation of transport		2.0*
neuromuscular synaptic transmission	2.9**	
neutral lipid metabolic process	-2.0*	
nuclear transcribed mrna catabolic process nonsense mediated decay	2.2*	
nucleobase containing small molecule metabolic process	2.2*	2.8**
nucleoside monophosphate metabolic process	2.6**	2.7**
nucleoside triphosphate metabolic process	2.7**	2.6**
organic anion transport	-1.8	-1.9*
organonitrogen compound metabolic process		1.9**
organophosphate metabolic process	2.1**	2.6**
phosphate containing compound metabolic process	1.8*	2.0**
positive regulation of biosynthetic process	1.8*	2.0**
positive regulation of cellular protein localization		2.1*
positive regulation of cell communication	1.8*	2.1**
positive regulation of cytokine production	2.6**	2.6**
positive regulation of establishment of protein localization		2.1*
positive regulation of gene expression	1.8*	2.2**
positive regulation of hydrolase activity		1.8*
positive regulation of immune system process	2.4**	2.5**
positive regulation of intracellular signal transduction	2.1*	2.4**
positive regulation of intracellular transport		2.1*
positive regulation of molecular function	2.1**	2.0**
positive regulation of multicellular organismal process	2.0*	2.2**
positive regulation of nf kappab transcription factor activity		2.6**
positive regulation of organelle organization		2.0*
positive regulation of response to stimulus	1.8*	2.0**
positive regulation of sequence specific dna binding transcription factor activity		2.6**
positive regulation of transport		1.8*
posttranscriptional regulation of gene expression		2.0*
protein folding	2.7**	3.3**
protein localization to endoplasmic reticulum	2.2*	
protein localization to membrane	2.3**	
protein targeting to membrane	2.2**	
purine containing compound metabolic process	2.5**	2.8**
regulated exocytosis		-2.0*
regulation of anion transport	1.9*	
regulation of apoptotic signaling pathway	2.6**	2.7**
regulation of cellular component biogenesis	2.1*	2.7**
regulation of cellular localization		2.4**
regulation of cellular protein localization		2.1*
regulation of cellular response to heat	2.8**	2.8**
regulation of cellular response to stress	2.4**	3.0**
regulation of cell death	2.1**	2.2**
regulation of cytokine production	2.5**	2.5**
regulation of cytoplasmic transport		2.0*
regulation of defense response	2.1*	2.5**
regulation of exocytosis		-1.9*
regulation of extrinsic apoptotic signaling pathway	2.4**	2.5**
regulation of immune response	2.5**	2.6**
regulation of immune system process	2.3**	2.4**
regulation of innate immune response	2.5**	2.7**
regulation of intracellular protein transport		2.0*
regulation of intracellular signal transduction		2.2**
regulation of intracellular transport	2.0*	2.7**
regulation of ion transport	1.9*	
regulation of membrane potential	2.2**	
regulation of mitochondrion organization	2.4**	
regulation of organelle organization		2.6**
regulation of protein localization		1.9*
regulation of protein modification by small protein conjugation or removal	2.6**	2.6**

regulation of protein modification process		1.9**
regulation of protein stability	2.4**	2.6**
regulation of receptor activity		-1.9*
regulation of response to cytokine stimulus	2.5**	
regulation of response to stress	2.0**	2.6**
regulation of rna stability	2.1*	
regulation of sequence specific dna binding transcription factor activity	2.7**	2.6**
regulation of transport	1.8*	2.2**
response to abiotic stimulus	2.1**	2.8**
response to alkaloid	2.6**	
response to endogenous stimulus	2.0**	
response to heat	2.8**	
response to nicotine	2.8**	
response to nitrogen compound	1.9*	
response to organic cyclic compound	1.7*	
response to oxidative stress	2.6**	2.8**
response to temperature stimulus	2.7**	
response to topologically incorrect protein	2.6**	2.7**
ribonucleoprotein complex biogenesis	2.1*	
ribosome biogenesis	2.4**	
rrna metabolic process	2.5**	
secretion		-2.1*
secretion by cell		-2.0*
sexual reproduction		2.0*
single organism behavior	2.1*	
skeletal muscle organ development	2.1*	
small gtpase mediated signal transduction	-1.9*	
synaptic signaling	2.3**	
synaptic transmission cholinergic	2.9**	
system process	2.2**	
translational initiation	2.4**	
transmembrane transport	1.9*	
vesicle mediated transport		-1.7*
viral life cycle	2.1*	2.9**

* $p<0.05$
** $p<0.01$

Supplementary Table 5. Genes used in qPCR validation, showing their fold change in RNAseq and qPCR

E2F1 target gene	Fold change RNAseq	Fold change qPCR
dad	1.11	3.42
drpr	1.28	1.59
siz	1.1	3.35
spi	1.11	2.08
DNApol-iota	1.18	1.94
csk	1.12	3.31
sals	1.2	1.76

Supplementary Table 6. Transcription factors enriched in the TyrRS-E196K brain transcriptome, in both up-and down-regulated gene lists.

Transcription factor	Gene symbol	E-score	Targets	Motifs
**Downregulated gene list**				
DNA replication-related element factor	Dref	11.113	77	12
cropped	crp	7.670	52	13
longitudinals lacking	lola	5.701	27	5
Zinc-finger protein interacting with CP190	CG7928	5.546	45	3
nautilus	nau	4.520	42	4
Heat shock factor	Hsf	4.121	37	8
Cyclic-AMP response element binding protein A	CrebA	4.010	25	10
Myocyte enhancer factor 2	Mef2	3.931	31	6
Hormone-receptor-like in 78	Hr78	3.794	48	7
tango	tgo	3.768	40	32
CG7786	CG7786	3.693	25	5
crocodile	croc	3.631	13	2
TBP-associated factor 1	Taf1	3.630	25	1
pannier	pnr	3.532	45	11
broad	br	3.476	13	2
Topoisomerase 2	Top2	3.473	19	2
jumeau	jumu	3.426	16	2
Chorion factor 2	Cf2	3.380	28	18
araucan	ara	3.367	23	4
Rfx	Rfx	3.322	13	2
p53	p53	3.226	27	5
senseless	sens	3.194	15	2
Hormone receptor-like in 39	Hr39	3.120	28	3
pangolin	pan	3.068	7	1
CG17829	CG17829	3.052	16	1
gemini	gem	2.780	10	1
stripe	sr	2.776	6	4
grain	grn	2.714	21	4
hamlet	ham	2.689	8	1
nejire	nej	2.578	6	1
scratch	scrt	2.560	35	2
schnurri	shn	2.521	10	1
**Upregulated gene list**				
slow border cells	slbo	4.816	54	18
Transcription factor IIB	TfIIB	4.070	30	4
grainy head	grh	3.976	39	3
wicked	wcd	3.923	12	1
Hormone receptor 3	Hr46	3.607	54	5
forkhead box, sub-group O	foxo	3.582	22	1
Cyclic-AMP response element binding protein B	CrebB-17A	3.557	72	24
snail	sna	3.403	45	7
E2F transcription factor	E2f	3.283	57	6
gemini	gem	3.260	30	2
erect wing	ewg	3.180	37	5
ovo	ovo	3.166	15	1
Mesoderm-expressed 2	Mes2	3.139	45	7
TATA binding protein	Tbp	3.134	51	4
Zn finger homeodomain 1	zfh1	3.129	42	2
Hormone receptor-like in 38	Hr38	2.987	23	2
Ptx1	Ptx1	2.984	42	3
cropped	crp	2.936	55	6
Heat shock factor	Hsf	2.856	38	3
brinker	brk	2.757	40	3
achintya	achi	2.719	33	2
cousin of atonal	cato	2.629	19	3
sloppy paired 2	slp2	2.614	6	1
Hormone receptor-like in 39	Hr39	2.558	34	1

pleiohomeotic	pho	2.508	17	1

E-score, enrichment score

Supplementary Table 7. GO terms enriched for the transcription factors in the TyrRS-E196K brain transcriptome

Term	Count	%	P-Value	Benjamini
positive regulation of transcription from RNA polymerase II promoter	15	39,5	4,6E-15	1,2E-12
regulation of transcription, DNA-templated	15	39,5	1,1E-11	1,5E-9
dendrite morphogenesis	11	28,9	2,3E-10	2,1E-8
transcription, DNA-templated	13	34,2	1,3E-9	8,7E-8
regulation of transcription from RNA polymerase II promoter	10	26,3	3,1E-9	1,7E-7
negative regulation of transcription from RNA polymerase II promoter	9	23,7	2,1E-8	9,3E-7
positive regulation of transcription, DNA-templated	8	21,1	9,4E-8	3,6E-6
muscle organ development	6	15,8	6,8E-6	2,3E-4
regulation of glucose metabolic process	7	18,4	2,1E-5	6,3E-4
transcription from RNA polymerase II promoter	5	13,2	6,4E-5	1,7E-3
neuron development	4	10,5	5,4E-4	1,3E-2
steroid hormone mediated signaling pathway	3	7,9	1,5E-3	3,4E-2
central nervous system development	4	10,5	2,4E-3	4,9E-2
imaginal disc-derived wing morphogenesis	5	13,2	5,3E-3	9,8E-2
compound eye development	4	10,5	8,1E-3	1,4E-1
compound eye morphogenesis	4	10,5	1,2E-2	1,9E-1
mesodermal cell fate determination	2	5,3	1,6E-2	2,3E-1
negative regulation of gene expression	3	7,9	1,7E-2	2,3E-1
sensory organ development	3	7,9	2,0E-2	2,5E-1
determination of muscle attachment site	2	5,3	2,9E-2	3,3E-1
peripheral nervous system development	3	7,9	3,1E-2	3,3E-1
cell death	2	5,3	3,9E-2	3,8E-1
cuticle development	2	5,3	4,8E-2	4,4E-1
neuroblast proliferation	2	5,3	5,7E-2	4,9E-1
transforming growth factor beta receptor signaling pathway	2	5,3	5,7E-2	4,9E-1
negative regulation of transcription, DNA-templated	3	7,9	5,8E-2	4,8E-1
RNA polymerase II transcriptional preinitiation complex assembly	2	5,3	6,7E-2	5,1E-1
ectoderm development	2	5,3	7,3E-2	5,3E-1
oogenesis	4	10,5	8,3E-2	5,7E-1
positive regulation of gene expression	2	5,3	8,5E-2	5,6E-1
glial cell migration	2	5,3	9,7E-2	6,0E-1

Supplementary Table 8. *Drosophila* stocks used throughout the paper

Stock genotype	Origin	Number
w1118	BDSC	3605
GMR-GAL4/CyO	BDSC	8605
nSyb-GAL4	M. Leyssen & B. Dickson (HHMI's Janelia Research Campus)	
Act5C-Gal4/Cyo,GFP	BDSC	derived from 4414
Act5C-Gal4/Cyo,Tb-RFP	BDSC	derived from 4414
R91H05-Gal4	BDSC	40594
w1118; P{UAS-E2f1.N}3B P{UAS-Dp.D}1-4b/TM6B,Tb1	BDSC	4770
w; P{UASp-GFP-E2f1.WT}4*	BDSC	34058

Supplementary Table 9. qPCR primers used in the different quantification assays

**Human**		
**Gene**	**Forward primer**	**Reverse primer**
RAD51L1	GCATTCTTATCTACTACCCTTTC	CTGCTATTTCAACCAGTCTTTCA
RAD51	GAGTTTGGTGTAGCAGTGGTAAT	GACAGGGAGAGTCGTAGATTTTG
RAD9A	TGAGATGTGCCTTGGAGAGGAGG	GCGAGTCGGTGTCTGAGAGTGTG
BRCA1	GAAACCAGTCTCAGTGTCCAACTCTCTA	GGTGATTTGTAACAATTCTTGATCTCCC
GAPDH	TGCACCACCAACTGCTTAGC	GGCATGGACTGTGGTCATGAG
HPRT1	TGACACTGGCAAAACAATGCA	GGTCCTTTTCACCAGCAAGCT
TBP	TGCACAGGAGCCAAGAGTGAA	CACATCACAGCTCCCCACCA
SDHA	TGGGAACAAGAGGGCATCTG	CCACCACTGCATCAAATTCATG
HMBS	GGCAATGCGGCTGCAA	GGGTACCCACGCGAATCAC
Drosophila melanogaster		
**Gene**	**Forward primer**	**Reverse primer**
dad	GACTGCGGTTACCAATCCAA	CAGTGGTGTGCACTTGTTTAAAAT
drpr	CGCCTGCGATATAAGCGTTC	GCAGGAGGAGTTATTCAGGCA
siz	TCAGTGTTTCCTCTGACCGA	AAAGCTTTCGCCAATTATAAAACCC
spi	CCCATTGCTTTGCGGTGAAG	GGCCTCTTGGGCAGGTAAG
DNApol-iota	GGCCAACATTCTACCCTCCC	CGCTCAAAAAGTCGCATCACA
csk	GCCTTGTCTTCACCAGCAAG	GGCAGTGTCGTAGGCAAAAA
trh	GCTACACTTGGCTCCAGACC	CCCGGTTGCTGATCACATAGT
sals	GAGTCCGAGGAGTCCGAAT	GAGTTGGGCGAGTCCTCTG
Act79B	CAAGGATCTGTATGCCAACAATG	GGTCAGCGATACCTGGATACATG
GAPDH	CAGCCCCGACATGAAGGT	CGATCTCGAAGTTGTCATTGATG
Dps13	GGGTCTGAAGCCCGACATT	GGCGACGGCCTTCTTGAT
PpL32	GCTAAGCTGTCGCACAAATGG	CGGCGACGCACTCTGTT
Rp49	TACAGGCCCAAGATCGTGAA	TCTCCTTGCGCTTCTTGGA
