# Supplementary material for: Transcriptional dysregulation by a nucleus-localized aminoacyl-tRNA synthetase associated with Charcot-Marie-Tooth neuropathy
Source: Nat Commun. 2019 Nov 6;10:5045. doi: 10.1038/s41467-019-12909-9 (PMC6834567; doi:10.1038/s41467-019-12909-9)
Supplement: Supplementary file 3 — Reporting Summary [file 41467_2019_12909_MOESM3_ESM.pdf]

nature research

Corresponding author(s): DBPR NCOMMS-19-06302A

Last updated by author(s): Sep 2, 2019

# Reporting Summary

Nature Research wishes to improve the reproducibility of the work that we publish. This form provides structure for consistency and transparency in reporting. For further information on Nature Research policies, see Authors & Referees and the Editorial Policy Checklist.

## Statistics

For all statistical analyses, confirm that the following items are present in the figure legend, table legend, main text, or Methods section.

| n/a | Confirmed | |
|---|---|---|
| ☐ | ☒ | The exact sample size ($n$) for each experimental group/condition, given as a discrete number and unit of measurement |
| ☐ | ☒ | A statement on whether measurements were taken from distinct samples or whether the same sample was measured repeatedly |
| ☐ | ☒ | The statistical test(s) used AND whether they are one- or two-sided<br>*Only common tests should be described solely by name; describe more complex techniques in the Methods section.* |
| ☒ | ☐ | A description of all covariates tested |
| ☐ | ☒ | A description of any assumptions or corrections, such as tests of normality and adjustment for multiple comparisons |
| ☐ | ☒ | A full description of the statistical parameters including central tendency (e.g. means) or other basic estimates (e.g. regression coefficient) AND variation (e.g. standard deviation) or associated estimates of uncertainty (e.g. confidence intervals) |
| ☐ | ☒ | For null hypothesis testing, the test statistic (e.g. $F$, $t$, $r$) with confidence intervals, effect sizes, degrees of freedom and $P$ value noted<br>*Give P values as exact values whenever suitable.* |
| ☒ | ☐ | For Bayesian analysis, information on the choice of priors and Markov chain Monte Carlo settings |
| ☒ | ☐ | For hierarchical and complex designs, identification of the appropriate level for tests and full reporting of outcomes |
| ☐ | ☒ | Estimates of effect sizes (e.g. Cohen's $d$, Pearson's $r$), indicating how they were calculated |

*Our web collection on statistics for biologists contains articles on many of the points above.*

## Software and code

Policy information about availability of computer code

| | |
|---|---|
| Data collection | RT-PCR data collection utilized StepOnePlus Real Time PCR system (Applied Biosystems) or ViiA 7 software version 1.2.2 (Applied Biosystems).<br>Western blot membrane exposure and data collection utilized FlourChem M (Proteinsimple) or the ImageQuant LAS4000 v1.2 (GE Healthcare).<br>Negative geotaxis was performed using the Flycrawler software (Peira scientific instruments).<br>Image collection was achieved using the ZEN 2011 SP7 v14.0.7.201 (Zeiss) software or Nikon Elements Advance Research 4. |
| Data analysis | RT-PCR data analysis utilized StepOne Software v2.2.2 (Applied Biosystems) or ViiA 7 Software version 1.2.2 (Applied Biosystems) and qbase+ version 3.1 (biogazelle).<br>Western blot analysis utilized Photoshop or imageJ v1.46r.<br>Statistical analysis was performed using GraphPad Prism version 7.01.<br>Negative geotaxis data was analyzed with Flycrawler analysis software (Peira scientific instruments).<br>RNA sequencing data was analyzed using Trimmomatic v0.32, FASTQC v0.11.2, Tophat v2.0.12, HTSeq v0.6.1, DESeq2 v1.14.1, and wgcna v1.51.<br>Transcription factor analysis was performed using Cytoscape v3.6.0 with the iRegulon v1.2 plugin.<br>To calculate and draw custom Venn diagrams the following online tool was used: http://bioinformatics.psb.ugent.be/webtools/Venn/.<br>R v3.3.1 was used for immunohistochemistry image quantification.<br>Giant fiber recordings were analysed using pCLAMP 10.3 software (Molecular Devices).<br>The effect sizes were calculated using the PlotsOfDifference webtool.<br>Maximum intensity projections of image z- stacks comprising the entire NMJ were analysed using the Fiji distribution of ImageJ50,51. All boutons were scored using the ImageJ Cell Counter plugin. The length of the neuronal arbour projecting on the muscle was determined using the ImageJ NeuronJ plugin. |

For manuscripts utilizing custom algorithms or software that are central to the research but not yet described in published literature, software must be made available to editors/reviewers. We strongly encourage code deposition in a community repository (e.g. GitHub). See the Nature Research guidelines for submitting code & software for further information.

## Data

Policy information about availability of data

All manuscripts must include a data availability statement. This statement should provide the following information, where applicable:

- Accession codes, unique identifiers, or web links for publicly available datasets
- A list of figures that have associated raw data
- A description of any restrictions on data availability

Source data are provided as a Source Data file. All data generated or analysed during this study are included in this published article (and its supplementary information files). The sequencing data are deposited at the GEO database (GSE125311). All other relevant data are available from the corresponding authors upon request.

# Field-specific reporting

Please select the one below that is the best fit for your research. If you are not sure, read the appropriate sections before making your selection.

☒ Life sciences ☐ Behavioural & social sciences ☐ Ecological, evolutionary & environmental sciences

For a reference copy of the document with all sections, see nature.com/documents/nr-reporting-summary-flat.pdf

# Life sciences study design

All studies must disclose on these points even when the disclosure is negative.

| | |
|---|---|
| Sample size | No sample size determination was performed. For the negative geotaxis assay, 10 repetitive measurements of a group of 10 flies was performed for at least 10 different groups to obtain reproducible results. To assess developmental lethality, at least 300 flies or pupae were counted, arising from 3 independent crosses to obtain reproducible results. |
| Data exclusions | The PCA and the hierarchical clustering of the regularized log counts revealed 3 outlier samples (nSyb-Gal4>+(11), nSyb-Gal4>TyrRS_WT(12), nSyb-Gal4>TyrRS_WTΔNLS (18)) which were removed from the final differential expression analysis, to avoid bias in the dataset.<br>For the negative geotaxis assay each group of 10 flies was measured 15 times and the average of the 10 fastest walking speeds was calculated. Groups that did not show any climbing behaviour or a wide spread between flies (>4secs) were excluded. |
| Replication | For the Drosophila experiments, three individual crosses were set up to replicate the findings. RNA sequencing analysis was performed on 4 individual biological replicates originating from individual crosses to account for variability within biological samples.<br>Experiments performed on cells were repeated three times on individual extractions and a representable image is shown. |
| Randomization | Data acquisition for the negative geotaxis assay was performed on an automated system where flies were blindly and randomly placed into the machine and tracked by the system. |
| Blinding | Data acquisition for the negative geotaxis assay was performed on an automated system where flies were blindly and randomly placed into the machine and tracked by the system. |

# Reporting for specific materials, systems and methods

We require information from authors about some types of materials, experimental systems and methods used in many studies. Here, indicate whether each material, system or method listed is relevant to your study. If you are not sure if a list item applies to your research, read the appropriate section before selecting a response.

### Materials & experimental systems

| n/a | Involved in the study | |
|---|---|---|
| ☐ | ☒ | Antibodies |
| ☐ | ☒ | Eukaryotic cell lines |
| ☒ | ☐ | Palaeontology |
| ☐ | ☒ | Animals and other organisms |
| ☒ | ☐ | Human research participants |
| ☒ | ☐ | Clinical data |

### Methods

| n/a | Involved in the study | |
|---|---|---|
| ☒ | ☐ | ChIP-seq |
| ☒ | ☐ | Flow cytometry |
| ☒ | ☐ | MRI-based neuroimaging |

## Antibodies

| | |
|---|---|
| Antibodies used | Rabbit polyclonal HRP antibody: Jackson ImmunoResearch laboratories, catalog number: 323-005-021;<br>Mouse monoclonal DLG antibody: Developmental Studies Hybridoma Bank, catalog number: 4F3 anti-discs large;<br>Mouse monoclonal TyrRS antibody: Abnova, catalog number: H00008565-M02;<br>Rabbit polyclonal TyrRS antibody: in house generated; |

| | |
|---|---|
| | Rabbit polyclonal GAPDH antibody: GeneTex, catalog number: GTX100118;<br>Mouse monoclonal α-Tubulin antibody: abcam, catalog number: ab7291;<br>Mouse monoclonal α-Tubulin antibody: Cell Signaling Technology, catalog number: #3873;<br>Mouse monoclonal Lamin Dm0 antibody: Developmental Studies Hybridoma Bank, catalog number: ADL67.10;<br>Mouse monoclonal Lamin A/C antibody: Cell Signaling Technology, catalog number: #4777;<br>Mouse monoclonal V5 antibody: Thermo Fisher Scientific, catalog number: R960CUS;<br>Rabbit polyclonal TRIM28 antibody: AbCam, catalog number: ab10484;<br>Rabbit monoclonal Flag antibody: Sigma-Aldrich, catalog number: F2555;<br>Rabbit polyclonal HA antibody: Abcam, catalog number: ab9110;<br>Mouse monoclonal E2F1 antibody for IP: AbCam, catalog number: ab4070;<br>Rabbit polyclonal E2F1 antibody for Western blot: AbCam, catalog number: ab112580;<br>Rabbit polyclonal acetylated-lysine antibody: Cell Signaling Technology, catalog number: #9441<br>Rabbit polyclonal HDAC1 antibody: Cell Signaling Technology, catalog number: #2062<br>Rabbit polyclonal SIRT1 antibody: Cell Signaling Technology, catalog number: #2310 |
| Validation | Rabbit polyclonal HRP antibody: PMID 18369958;<br>Mouse monoclonal DLG antibody: PMID 28744001;<br>Mouse monoclonal TyrRS antibody: https://www.antibodypedia.com/gene/17005/YARS/antibody/322069/H00008565-M02;<br>Rabbit polyclonal TRIM28 antibody: http://www.abcam.com/kap1-antibody-ab10484.html;<br>Mouse monoclonal E2F1 antibody for IP: http://www.abcam.com/e2f1-antibody-kh95-chip-grade-ab4070.html;<br>Rabbit polyclonal E2F1 antibody for Western blot: http://www.abcam.com/e2f1-antibody-ab112580.html;<br>Rabbit polyclonal acetylated-lysine antibody: https://www.cellsignal.com/products/primary-antibodies/acetylated-lysine-antibody/9441;<br>Rabbit polyclonal GAPDH antibody: https://www.antibodypedia.com/gene/3923/GAPDH/antibody/169644/GTX100118;<br>Mouse monoclonal α-Tubulin antibody: http://www.abcam.com/alpha-tubulin-antibody-dm1a-loading-control-ab7291.html;<br>Mouse monoclonal α-Tubulin antibody: https://www.cellsignal.com/products/primary-antibodies/a-tubulin-dm1a-mouse-mab/3873;<br>Mouse monoclonal Lamin Dm0 antibody: PMID 25512562;<br>Mouse monoclonal Lamin A/C antibody: https://www.cellsignal.com/products/primary-antibodies/lamin-a-c-4c11-mouse-mab/4777;<br>Mouse monoclonal V5 antibody: https://www.thermofisher.com/order/catalog/product/R960CUS;<br>Rabbit polyclonal TyrRS antibody: PMID 25284223;<br>Rabbit monoclonal Flag antibody: https://www.sigmaaldrich.com/catalog/product/SIGMA/F2555?lang=en&region=US;<br>Rabbit polyclonal HA antibody: http://www.abcam.com/ha-tag-antibody-chip-grade-ab9110.html.<br>Rabbit polyclonal HDAC1 antibody: https://www.cellsignal.com/products/primary-antibodies/histone-deacetylase-1-hdac1-antibody/2062?site-search-type=Products<br>Rabbit polyclonal SIRT1 antibody: https://www.cellsignal.com/products/primary-antibodies/sirt1-antibody/2310 |

## Eukaryotic cell lines

Policy information about cell lines

| | |
|---|---|
| Cell line source(s) | The HEK293T (human embryonic kidney) cell line was purchased from ATCC (Manassas, VA, USA).<br>The lymphoblast cell lines were derived from peripheral blood drawn from CMT patients carrying the E196K or G41R mutations, and control individuals. |
| Authentication | The HEK293T cell line was authenticated by morphology check by microscope. |
| Mycoplasma contamination | The HEK293T cell line was tested negative for mycoplasma contamination.<br>Lymphoblast lines were tested negative for mycoplasma contamination before use. |
| Commonly misidentified lines<br>(See ICLAC register) | *Name any commonly misidentified cell lines used in the study and provide a rationale for their use.* |

## Animals and other organisms

Policy information about studies involving animals; ARRIVE guidelines recommended for reporting animal research

| | |
|---|---|
| Laboratory animals | For the fly experiments, Drosophila melanogaster was used of the Canton S strain. Female flies were used for all the experiments. This study was approved by the ethical committee for biomedical experiments with animals of the University of Antwerp (EDC 2014-02). |
| Wild animals | The study did not involve wild animals. |
| Field-collected samples | The study did not involve samples collected from the field. |
| Ethics oversight | No ethical approval was required as no new patient samples was obtained and no vertebrate animals were used for this study. |

Note that full information on the approval of the study protocol must also be provided in the manuscript.
